# Comparison of Two Water Color Algorithms: Implications for the Remote Sensing of Water Bodies with Moderate to High CDOM or Chlorophyll Levels

**DOI:** 10.3390/s23031071

**Published:** 2023-01-17

**Authors:** Martha Otte Burket, Leif G. Olmanson, Patrick L. Brezonik

**Affiliations:** 1 Department of Civil, Environmental, and Geo-Engineering, University of Minnesota, Minneapolis, MN 55455, USA; 2 Department of Forest Resources, University of Minnesota, Saint Paul, MN 55455, USA

**Keywords:** dominant wavelength, hue angle, water color, colored dissolved organic matter (CDOM), chlorophyll, Forel–Ule index, reflectance spectra, Sentinel-2, Sentinel-3, Landsat 8

## Abstract

The dominant wavelength and hue angle can be used to quantify the color of lake water. Understanding the water color is important because the color relates to the water quality and its related public perceptions. In this paper, we compared the accuracy levels of two methods in calculating dominant wavelength and hue angle values using simulated satellite data calculated from in situ reflectance hyperspectra for 325 lakes and rivers in Minnesota and Wisconsin. The methods developed by van der Woerd and Wernand in 2015 and Wang et al. in 2015 were applied to simulated sensor data from the Sentinel-2, Sentinel-3, and Landsat 8 satellites. Both methods performed comparably when a correction algorithm could be applied, but the correction method did not work well for the Wang method at hue angles < 75°, equivalent to levels of colored dissolved organic matter (CDOM, *a*_440_) > ~2 m^−1^ or chlorophyll > ~10 mg m^−3^. The Sentinel-3 spectral bands produced the most accurate results for the van der Woerd and Wernand method, while the Landsat 8 sensor produced the most accurate values for the Wang method. The distinct differences in the shapes of the reflectance hyperspectra were related to the dominant optical water quality constituents in the water bodies, and relationships were found between the dominant wavelength and four water quality parameters, namely the Secchi depth, CDOM, chlorophyll, and Forel–Ule color index.

## 1. Introduction

The multi-spectral imagery from space-borne Earth observation platforms can be used to monitor inland water resources for a variety of water quality metrics, including the levels of chlorophyll (CHL) [1,2], colored-dissolved organic matter (CDOM) [3,4,5,6], and water clarity, measured as the Secchi depth (SD) [7,8,9,10]. The monitoring of water quality using satellite imagery has the advantages of allowing greater temporal and spatial scopes than ground-based measurements. Traditionally, classifications of surface water pixels from raw top-of-atmosphere (TOA) measurements into biophysical units such as the CHL (mg/m^3^) are developed from mathematical relationships between the remotely sensed imagery and associated data for the corresponding water quality metric measured in the field.

The water color, determined by various dissolved and suspended biogeochemical constituents in the water column, is related to the human perceptions of water quality. The color itself, thus, is a water quality metric. The hue angle, α, and dominant wavelength, λ_d_, which are quantitative measures of surface water color, have been calculated from satellite multi-spectral imagery data. Two sets of procedures were developed for this purpose, defined here as (1) the vdWW or van der Woerd and Wernand method [11,12] and (2) the Wang method [13,14]. The Wang method was initially applied to Lake Taihu (a large lake in China), while the vdWW method was initially applied to the North Sea and Dutch water bodies (coastal and inland) [11,13]. Both procedures have been used in recent studies to examine trends in water color in lakes over time and across regions (e.g., [15,16,17]), but a comparison of the accuracy of the two methods using different sensors has not been reported.

As the research about the relationships between water color and water quality metrics continues to develop, it will be important to ensure that the proposed methods are appropriate. For this reason, the goal of this research is to compare the vdWW and Wang calculations for the hue angle and dominant wavelength using simulated imagery for three low Earth orbiting satellite sensors (Sentinel-2 Multispectral Instrument (MSI), Sentinel-3 Ocean Land Color Instrument (OLCI), and Landsat 8 Operational Land Imager (OLI)) based on more than three hundred hyperspectra, mostly from more than three hundred glacial lakes across Minnesota and Wisconsin, USA, which differ in their optical properties. Additionally, the relationships between λ_d_ and the water quality parameters (CDOM, CHL, and SD) are explored.

## 2. Background

### 2.1. Calculation of the Hue Angle and Dominant Wavelength

**Figure 1:**
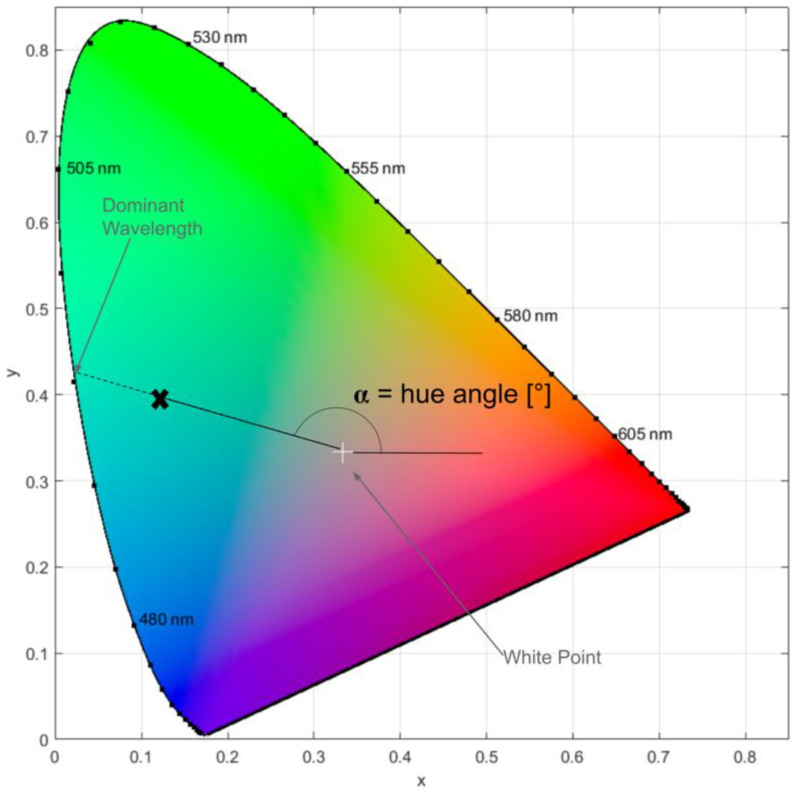
Diagram of the CIE 1931 color space with key terms (dominant wavelength, hue angle) noted. The bold X represents the location of an arbitrary color, the dominant wavelength of which is determined by the straight line extending from the white point through it to the edge of the color space. The x- and y-values on the axes are chromaticity coordinates. Plot created using code adapted from [15,16].

The calculation of α and λ_d_ (λ_d_) is a multistep process. First, the tristimulus values (X, Y, and Z) are calculated. The tristimulus values correspond to the amounts of primary colors needed to produce a certain color stimulus [18]. The tristimulus values can be converted into chromaticity coordinates, which allow a color to be plotted with x- and y-coordinates in a two-dimensional color space, such as the International Commission on Illumination (CIE) 1931 color space (Figure 1)). The white point (WP) corresponds to the color white. To find λ_d_ for a given color sample, a line is extended from the WPWPWthe through a given color point (e.g., X in Figure 1) to the edge of the color space. The intersection of this line with the curve bounding the edge of the color space corresponds to the value of λ_d_, which characterizes a color’s hue and was originally defined by the CIE as the wavelength of monochromatic light that evokes an identical perception of the hue of a multispectral light source.

The hue angle (α) (Figure 1) is another measure of water color. The hue angle describes the angle between the horizontal axis and the color point, whereby α is inversely (and non-linearly) related to λ_d_ via a lookup table (Figure S1). This paper mostly uses λ_d_ to describe water color because we think it has a more intuitive meaning than α, but α is also used to facilitate a clear link to past research and because α is used to calculate λ_d_.

Two main methods have emerged for calculating α and λ_d_ from satellite data. The first method, the Wang method, was introduced by Wang et al. [13] and used with MODIS imagery to calculate λ_d_ from computed α values for Lake Taihu (the third largest lake in China). The method was applied to 13 satellite images and 73 field reflectance measurements. The second method, the vdWW method, published by van der Woerd and Wernand [11,12], focused on calculating α, and its accuracy was evaluated using 603 hyperspectral measurements from coastal and inland water bodies. Both methods, thus, consist of an initial calculation of α. In the Wang method, the calculations are performed in the same way for every satellite sensor using standard equations from the CIE. In the vdWW method, the calculations are performed for each satellite to account for the specific spectral locations of each satellite’s bands. In both methods, α is corrected to account for systematic errors that arise from calculating it with satellite data instead of hyperspectral data. The vdWW method introduced the idea of correcting α, while the MODIS corrections were added to the Wang method in 2018 [14]. However, as far as we know, coefficients to correct the Wang method have not been published for the satellite sensors used in this study.

### 2.2. Applications of Water Color Measurements

The implementation of Wang and vdWW calculations has allowed researchers to classify and map the colors of water bodies across large regions. Lehmann et al. [15] calculated the λ_d_ values for λ_d_ for 1486 lakes in New Zealand, while and Giardino et al. [16] calculated the λ_d_ values for 170 Italian lakes using the vdWW method. Large datasets have also been used with the Wang method to calculate λ_d_ values λ_d_ for lakes [17] and rivers [19] over time in the United States.

Furthermore, the α and λ_d_ can be used to calculate the Forel–Ule Index (FUI). The FUI visually matches the water color to one of 21 different colors ranging from blue to green to brown [20] (pp. 415–416). Wernand and van der Woerd [21] used aquatic remote sensing reflectance (*R_rs_*) measurements to calculate FUI values, and this work was extended by Novoa et al. [22], who provided a lookup table connecting the hue angle α to the FUI. Wang et al. [13] used the Novoa et al. method to calculate the FUI, which they then used [14] to evaluate the trophic status of large inland water bodies across the globe. Pitarch et al. [23] also used α to determine the FUI for global oceans. The FUI (determined visually) has been used since the late 1800s, and a substantial historical database for oceanic FUI values has been developed [21,24]. Thus, calculating the FUI with satellite imagery can help connect historical measurements and present conditions [21].

The water color is affected by the levels of inorganic suspended matter, phytoplankton, and colored dissolved organic matter (CDOM) [25,26]. For this reason, the water color has been related to various water quality metrics, although the success rates vary. For example, Wernand et al. [24] reported that trends of ocean surface CHL levels could be reconstructed from historical FUI data, but Malthus et al. [27] were unable to find a straightforward inverse relation between the color (quantified as the FUI) and the CHL, Secchi depth (SD), or suspended matter. Although simple relationships may be challenging to find, van der Woerd and Wernand [12] suggested that α could potentially be linked to the CHL level if the CDOM value was also known.

Several other potential uses of water color (measured as λ_d_ or α) parameters have been investigated. Zhao et al. [28] used α to identify water bodies with anomalous colors due to industrial, urban, or agricultural pollution; λ_d_ has also been connected to land use [15] and latitude [16]. Additionally, West et al. [29] noted that the water quality is linked to human perceptions of water bodies via clarity and color. The perceptions of color are then related to policies, economics, and cultural and spiritual values [29].

## 3. Materials and Methods

### 3.1. Satellite Sensors

**Figure 2:**
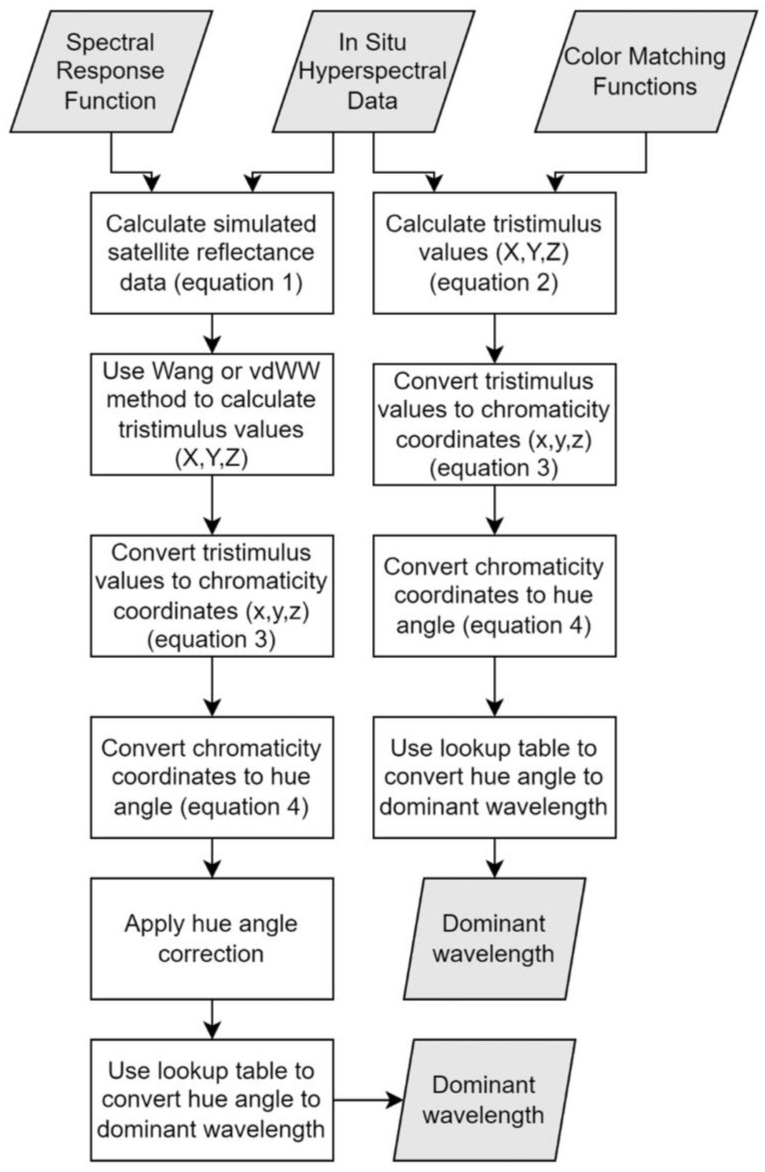
Flowchart of the calculation steps used to convert the hyperspectral data to the dominant wavelength, λ_d_.

Satellite data on water bodies typically are obtained by multispectral sensors that collect data within specific bands, which include the visible regions of the electromagnetic spectrum that are useful for water measurements. Humans perceive color within the wavelength range of approximately 400 to 700 nm [30], and bands within these wavelengths can be used to calculate α and λ_d_ using the vdWW and Wang methods. This paper focuses on three commonly used satellite sensors: MSI, OLCI, and OLI. Section 3.2, Section 3.3 and Section 3.4 describe the calculations used for this paper. A summary of the calculations is provided in Figure 2.

### 3.2. Study Region and Dataset

**Figure 3:**
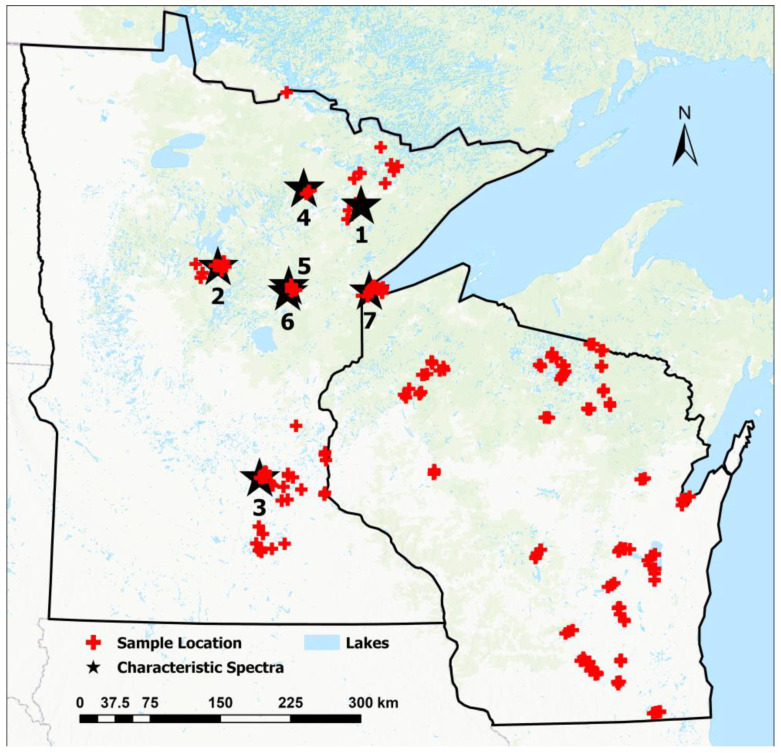
Locations of MNWI hyperspectral measurements. Numbered stars 1–7 are the locations of sites with hyperspectra discussed in Results Section 4.1 and shown in a later figure.

The in situ water quality and reflectance data were collected between 2013 and 2019 in the months between May and October. In total, 325 reflectance hyperspectra are in the dataset, hereafter referred to as the MNWI dataset. The data for the Minnesota lakes were collected by the authors, and the Wisconsin data were collected by the Bureau of Water Quality at the Wisconsin Department of Natural Resources [31]. The Wisconsin and a portion of the Minnesota data are part of a large global dataset [32]. Figure 3 shows the locations of the data collection sites.

The hyperspectral reflectance data were collected and processed with a system consisting of two Ocean Optics spectroradiometers (USB-2000 for WI and USB-2000+ for MN) with attached fiber-optic cables, a Spectralon calibration panel, and a laptop computer with software to record and process the data using methods described by Brezonik et al. [33]. At each site, 5–6 calibration scans were taken, followed by 5–7 scans just below the water surface. The software averaged at least 10 measurements (for high-CDOM water bodies), with the default being 25 measurements from each scan. A macro from the University of Nebraska’s Center for Advanced Land Management Information Technologies (CALMIT) was used to resample the original spectra collected at 0.4 nm intervals to 1 nm intervals from 400 to 900 nm.

The CHL and CDOM values were measured on Minnesota lakes with methods described by Brezonik et al. [34] and Griffin et al. [35]. The Wisconsin Department of Natural Resources used U.S. Environmental Protection Agency Method 445.0 [36] for CHL and a method described by Mitchell et al. [37] for CDOM values.

### 3.3. Simulated Satellite Data

The hyperspectral data were available at 1 nm intervals for wavelengths between 400 and 900 nm. Equation (1) was used to convert the hyperspectral data to the simulated *R_RS_* ($\hat{R}$) for particular satellite sensor bands:

(1) $$\hat{R}=\frac{\sum_{\lambda =\lambda_{min}}^{\lambda_{max}}SRF\left(\lambda \right)*R\left(\lambda \right)}{\sum_{\lambda =\lambda_{min}}^{\lambda_{max}}SRF\left(\lambda \right)}$$

where *R(λ)* is the measured hyperspectral reflectance, *SRF(λ)* is a spectral response function, and λ_min_ and λ_max_ are the minimum and maximum wavelengths where data for both *R(λ)* and *SRF(λ)* are available. When implementing Equation (1), the spectral response function (*SRF*) is specific for a band in a given satellite sensor, and the calculated *R* corresponds to that satellite band. The *SRF*s for Sentinel-3 had an average discretization of 0.1 nm, and to make the discretization consistent with the 1 nm discretization of the MNWI data, the satellite *SRF*s were resampled to a 1 nm discretization rate using linear interpolation. The *SRF* for Sentinel-3 band 1 had values for wavelengths outside of the range of the MNWI data. The *SRF* wavelengths for band 1 were defined between 387.7 nm and 411.3 nm, and the MNWI data were not measured below 400 nm. For this case, Equation (1) was implemented only for the *SRF* values within the range of available MNWI data. Equation (1) was used to calculate the reflectance values for Landsat 8, Sentinel-2, and Sentinel-3 bands, referred to here as simulated satellite data.

### 3.4. Calculation of Chromaticity Coordinates

The vdWW method calculated the tristimulus values *X*, *Y*, and *Z* by convolving *R_RS_* with a color-matching function (CMF). The relationship is given by Equation (2), which we used to convert the hyperspectral data to tristimulus values:

(2) $$T=\overset{710\;nm}{\underset{i=400\;nm}{\sum }}R_{RS}\left(\lambda \right)\bar{t}\left(\lambda \right)\Delta \lambda$$

where *T* represents a generic tristimulus value (i.e., *X*, *Y*, or *Z*), $\bar{t}\left(\lambda \right)$ represents the CMFs, and λ represents the wavelength. The CIE 1931 2-degree CMFs were used, consistent with the previous work [11,27].

Equation (2) cannot be used directly on satellite data, and for this process the vdWW and Wang calculations were used. The vdWW method derived coefficients for a given satellite that allow for the evaluation of Equation (2) by linearly interpolating between the available reflectance values. The vdWW method, thus, results in unique coefficients for a given satellite based on its unique band centers. To calculate the tristimulus values using vdWW, each available visible reflectance band is multiplied by a pre-calculated coefficient. The detailed methodology is available in [11,12].

The Wang method [13] uses an expression derived by a CIE commission. To apply this expression to satellite reflectance, red (R), green (G), and blue (B) bands must be selected. The standardized CIE wavelengths for these bands are R = 700 nm, G = 546.1 nm, and B = 435.8 nm, respectively [38]. The bands centered closest to these values must be selected to correctly apply the CIE formula. For the Wang method, the RGB bands are multiplied by pre-calculated coefficients from the CIE to calculate the tristimulus values, and the same coefficients are used regardless of the satellite sensor. The method is described in detail by Wang et al. [13].

To summarize, the tristimulus values were calculated directly from hyperspectral data and indirectly for the three satellite sensors using the vdWW and Wang methods, and the computed tristimulus values were then converted to chromaticity coordinates (x, y, and z) using Equation (3):

(3) $$t=\frac{T}{X+Y+Z}$$

where *t* represents a generic chromaticity coordinate (i.e., x, y, or z). The chromaticity coordinates correspond to a color located in the CIE color space. The location in the color space can also be represented using the hue angle, α:

(4) $$\alpha =arctan\left(y-y_{w},x-x_{w}\right)modulus2\pi$$

where *x_w_* = *y_w_* = 1/3 are the chromaticity coordinates of the white point. A correction can be applied to the calculated α to account for systematic errors resulting from the use of satellite data instead of hyperspectral data. In the vdWW method, α is corrected using a fifth-order polynomial [11,12] derived using an IOCCG synthetic dataset [26]. The same method of deriving the correction polynomial was adopted by Wang et al. [14] for the MODIS satellite, but correction polynomials were not derived for other satellite sensors. Consequently, we derived correction equations using the IOCCG data and the procedures described by van der Woerd and Wernand [11,12] and Wang et al. [14] for the three sensors used in this study. This process is discussed further in Section 3.5.

After α is calculated, it can be converted to λ_d_ using a lookup table; see [15] for details on this process, which can be accomplished in MATLAB using functions from the “color toolbox” [18,39]. We also used the α values for the hyperspectral data to compute the FUI values using the method and lookup table described by Novoa et al. [22].

The processdures described above were used to calculate λ_d_ values directly from hyperspectral data and indirectly from simulated satellite data (Landsat 8, Sentinel-2, and Sentinel-3) using the vdWW and Wang methods. The Wang method calculations were done with and without correction. In total, λ_d_ was calculated once directly from hyperspectral data, three times using the vdWW method, three times using the Wang method without corrections, and three times using the Wang method with corrections (in each case, once for each satellite). The values of λ_d_ and α calculated directly from the in situ hyperspectral data (λ_d,hyp_ and α_hyp_, respectively) were considered the “true” values of these variables.

### 3.5. Deriving Corrections for Wang Method

Systemic errors occur in hue angle (α) calculations based on satellite data, and van der Woerd and Wernand [11,12] used the IOCCG synthetic dataset to derive corrections for these errors. They found the errors in α measurements by subtracting the simulated satellite α value (in degrees) from the hyperspectral hue angle (α_hyp_). In this approach, α_hyp_ is considered the “true” value. We also used this approach with the IOCCG dataset to find the errors for Wang method α values for our dataset. The hue angle error (found by subtracting the simulated satellite α from α_hyp_) was calculated as an intermediate step when deriving the corrections (Figure S2). Similar to the findings for the vdWW method, we found that the error was fit by a fifth-order polynomial equation. For each satellite, the polynomial was fit over the range of 30° to 230°. The polynomial equation was used to calculate a correction, Δ, to add to the calculated α, where Δ is defined as:

(5) $$\Delta =c_{5}a^{5}+c_{4}a^{4}+c_{3}a^{3\;}+c_{2}a^{2}+c_{1}a+c$$

where a was calculated from the simulated satellite hue angle (α, in degrees) as:

(6) $$a=\frac{\alpha -30}{100}$$

**Figure 4:**
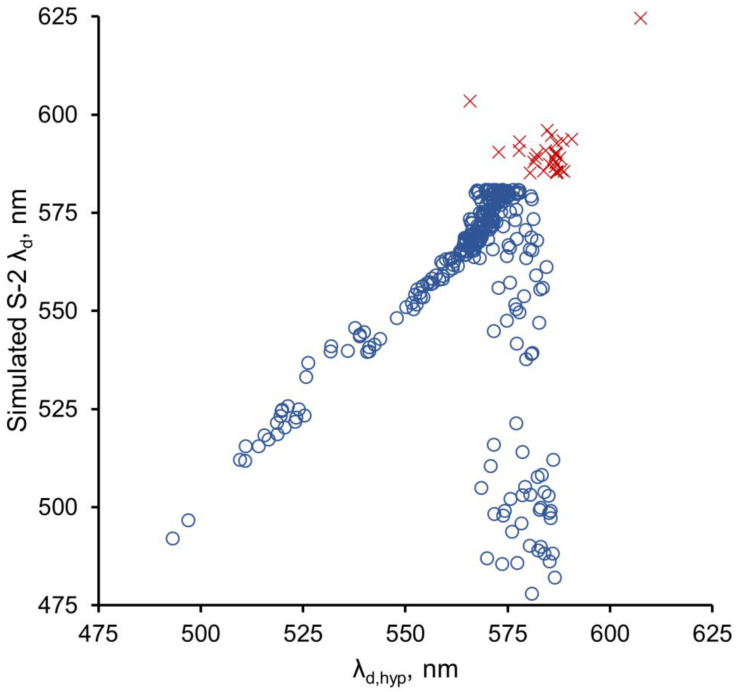
Wang method results for the S-2 MSI sensor for α corrections applied to angles between 30° and 230° (**○**); **×** represents uncorrected values outside the range where corrections were applied. Similar results were obtained for the S-3 OLCI and Landsat 8 OLI sensors.

This definition of the polynomial term “a” was used only for the Wang method. It differs from the definition used in the vdWW method by including the constant value 30 to center the data and resolve a badly conditioned error from the fitting software. Because of the poor performance of the Wang method for hue angles lower than 75° (Figure 4), the coefficients for the Δ equations for the Wang method were applied only over the range of 75° to 230°, and the calculated Δ values were added to the initial values of α to derive new corrected values. In contrast, the range of α corrections in the vdWW method is 30° to 230°.

The IOCCG dataset used to derive the corrections contained hue angles primarily above 75°, and this value, thus, was chosen as the lower limit for applying the hue angle corrections for the Wang method.

### 3.6. Statistical Methods

The calculated values of λ_d_, α, and FUI and the measured values of water quality parameters were assembled into Excel 2016 spreadsheets. The descriptive statistics, including the mean, median, interquartile values, mean absolute differences (MADs), mean absolute percentage differences (MAPDs), and bias, were calculated in Excel, and the distributional statistics were computed in JMP Pro 16 (SAS Institute, Inc., Cary, NC, USA). The bias was calculated as the sum of differences between a given method and the corresponding values from the hyperspectral measurements divided by the number of measurements. Simple regressions were performed in Excel, and multiple regressions in JMP 16. The histograms of the distribution of all λ_d_ and α values were skewed toward high λ_d_ and low α values. In all cases, however, the values were broadly distributed across the entire range for each variable. We did not transform the data for the statistical analyses because we were primarily interested in describing the linearity of the relationships between the two methods of calculating λ_d_. Similarly, the water quality variables (SD, CHL, CDOM, TSS) were skewed toward low values, but we did not transform those data because we were primarily interested in examining plots of these variables versus λ_d_.

## 4. Results

### 4.1. Characteristics of Reflectance Spectra

We calculated α_hyp_ and λ_d,hyp_ for 325 in situ reflectance spectra measured on 106 water bodies (mostly lakes and a few large rivers; Figure 3). The calculated λ_d,hyp_ values ranged from 493.2 nm to 607.4 nm, with an average of 567.5 nm (Table 1). Similarly, the α_hyp_ values ranged from 1.79° to 173.36° (average of 63.3°). The minimum λ_d,hyp_ was for Sabin Lake (St. Louis County, MN, USA), a deep, highly transparent lake in an abandoned iron mine pit (Secchi depth = 19.2 m), and the maximum was for Section Eleven Lake (Itasca County, MN, USA), a small bog lake with very high humic coloring. Conversely, Section Eleven Lake had the minimum α_hyp_, while Sabin Lake had the maximum α_hyp_. As described in Section 2.1, the relationship between α and λ_d_ is inverse (but nonlinear; Figure S1). The calculated FUI values for the water bodies ranged from 5 (blue-to-greenish-blue range of the index [20] (pp. 415–416)) for Sabin Lake to 21 (brown; the maximum value for the index) for the CDOM-rich Section Eleven Lake. The mean FUI of 13 for the 325 water bodies was in the green-yellow range of the index [20] (pp. 415–416) and suggested that on average the water bodies are optically dominated by a mixture of chlorophyll and CDOM.

**Figure 5:**
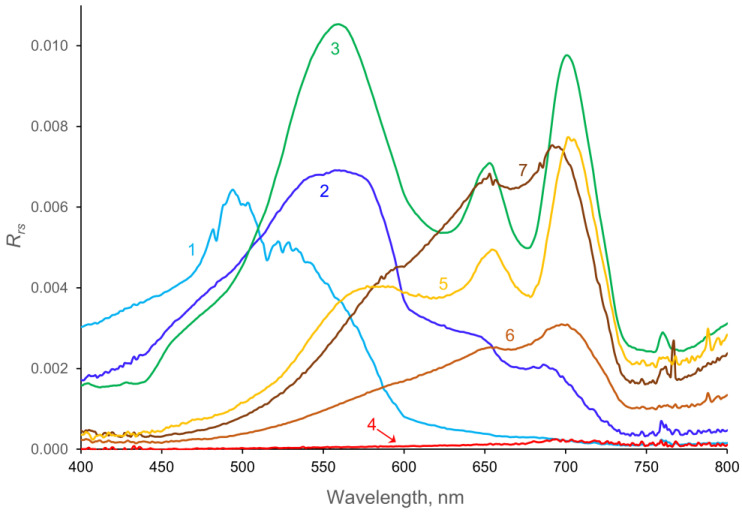
Reflectance spectra for water bodies dominated by various optical properties: (1) nearly pure water (Sabin Lake); (2) clear, oligotrophic water (Woman Lake); (3) chlorophyll (Halstead’s Bay); (4) CDOM (Section Eleven and Johnson Bog); (5) CDOM and chlorophyll (Big Sandy Lake); (6) CDOM and SM (Flowage Lake); (7) SM and CDOM (St. Louis River).

The reflectance spectra comprise a wide variety of shapes that generally depend on the relative abundances of three important “optical” constituents, namely the plant pigments (especially CHL), suspended matter (SM), and CDOM (Figure 5, Table S1), and cover a wide range of FUI values. The spectrum for Sabin Lake (the deep, very clear iron mine pit) has a reflectance maximum near 500 nm, a secondary maximum around 525 nm, and a shape close to that for pure water [39]. A more typical spectrum of a low-CDOM, oligotrophic water body (Woman Lake, FUI = 9, line 2) has a reflectance maximum near 560 nm, similar to that for the chlorophyll-dominated, eutrophic Halstead’s Bay in Lake Minnetonka (FUI = 12, line 3). However, the oligotrophic lake has only a very small reflectance trough at 670 nm (characteristic of chlorophyll absorption) and lacks the reflectance peak at 705 nm characteristic of scattering by phytoplankton cells. In contrast, Halstead’s Bay has a pronounced trough at 670 nm and a peak at 705 nm. The secondary minimum in the spectrum of chlorophyll-dominated Halstead’s Bay is characteristic of phycocyanin absorbance trough at 620 nm and indicates that cyanobacteria are present in the lake.

The four spectra labeled CDOM (4–7) all have low reflectance in the blue region (<500 nm) because of high CDOM absorbance. The very low spectra for Johnson Bog and Section Eleven Lake (line 4) represent water bodies that are optically dominated by very high CDOM values (*a*_440_ > 20 m^−1^), which absorb nearly all incoming light, leaving little to be reflected, even at long wavelengths. Line 5 (Big Sandy Lake, FUI = 16–19) contains water with both high CDOM and high chlorophyll levels, resulting in low reflectance in the blue region and a 670 nm trough and 705 nm peak characteristic of chlorophyll. The two spectra labeled SM and CDOM are for water bodies with high levels of suspended matter (largely clay and mineral particles) and high levels of CDOM (FUI = 20). They have low reflectance at wavelengths < 500 nm, characteristic of CDOM, and peaks at 705 nm, indicative of light scattering by suspended matter. Both have only small reflectance troughs at 670, indicating that the chlorophyll had little influence on their spectral properties.

### 4.2. vdWW and Wang Results and Comparison to Hyperspectral Values

**Figure 6:**
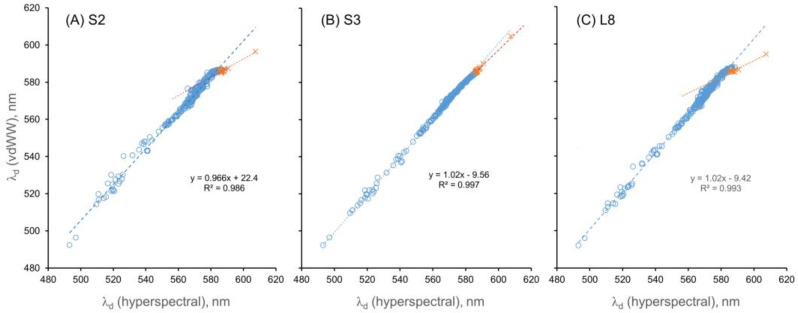
The calculated λ_d_ values using the vdWW method versus λ_d,hyp_ for the three sensors. The blue circles represent data points that were corrected; the orange (×) data points represent values where corrections could not be applied.

The vdWW method (using corrected α values) produced λ_d_ values with high accuracy (i.e., strong agreement with the corresponding λ_d,hyp_ values and low error rates across most of the range of λ_d,hyp_ in the dataset (Figure 6, Table 2)). The R^2^ values for the relationship between λ_d_ and λ_d,hyp_ were ≥ 0.99 for all three sensors, and the slopes of the best-fit lines were close to unity (0.97–1.02).

**Figure 7:**
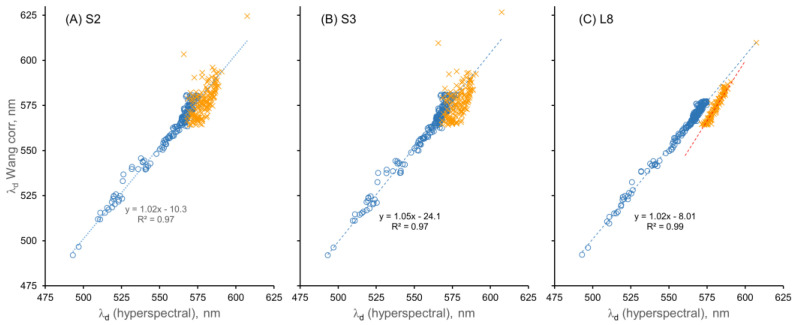
Calculated λ_d_ values using the Wang method versus λ_d,hyp_ after α correction for the three sensors. The blue circles represent data points where hue angle was corrected; the orange (×) data points represent values where corrections were not applied.

In contrast, the dominant wavelength (λ_d_) calculated by the Wang method using uncorrected α values gave much poorer fits (R^2^ = 0.78–0.95), with strong evidence of curvature in the relationships between λ_d_ and λ_d,hyp_ (Figure S3). The polynomial coefficients used to correct the α values for the Wang method (Table 3) allowed the calculation of λ_d_ values using corrected α values (Figure 7), similar to the vdWW approach. The linearity of the relationship between the Wang-calculated λ_d_ values for all three sensors and the λ_d,hyp_ values increased substantially for the corrected α values, as did the R^2^ values for the relationships (Table 2). The increased linearity and R^2^ values indicate that correcting the α values increased the accuracy of the Wang method. However, the corrections for the Wang method were applied only for α values of 75° to 230° (472.9 nm to 585.0 nm), in contrast to the range of 30–230° used in the vdWW method, because α was overestimated and λ_d_ was underestimated for low hue angle values (30–75°) when corrections were applied in the Wang method (Figure 4). Consequently, the high R^2^ values (0.97–0.99) of the Wang λ_d_–λ_d,hyp_ relationships for corrected α values do not apply across the entire range of λ_d_ values in the dataset, i.e., they do not apply at λ_d,hyp_ > 564.2 nm (α < 75°).

The regressions of the corrected Wang λ_d_ values versus the vdWW λ_d_ values for the three sensors (S-2, S-3, and L8; plots not shown) yielded high R^2^ values (0.98, 0.97, and 0.99, respectively), with the slopes of the relationships being close to unity (1.07, 1.02, and 0.99, respectively) and with low error rates (MADs of 2.37, 2.63, and 1.02 nm and MAPDs of 0.42, 1.37, and 0.48%, respectively); the vdWW results were the reference (assumed to be correct) values in each case. The bias values were small (0.8, 2.0, and 0.1 nm, respectively; L8 was especially low). Consequently, both methods worked well for water bodies for which the calculated α values could be corrected. For the vdWW method, this covered nearly the entire range of water bodies in the MNWI dataset, but α could not be corrected for lakes with moderate chlorophyll (>~10 mg m^−3^) or CDOM (>~2 *a*_440_ (m^−1^); relatively low α and high λ_d_) levels for the Wang method, and the method, thus, worked well only for ~55% of the water bodies in the MNWI dataset.

### 4.3. Dominant Wavelength and Water Quality Relationships

**Figure 8:**
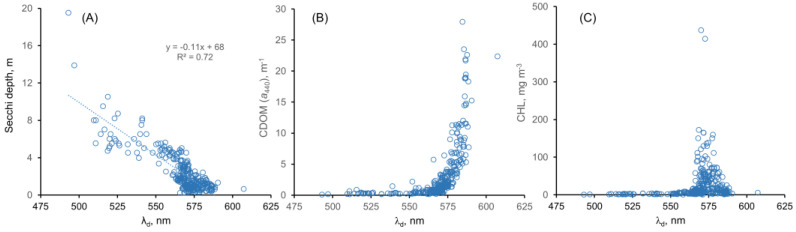
Relationships between some water quality metrics and λ_d,hyp_: (**A**) Secchi depth; (**B**) CDOM; (**C**) CHL.

**Figure 9:**
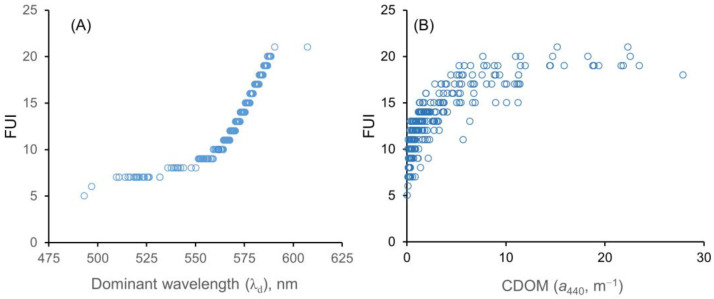
(**A**) The Forel–Ule index (FUI) values increase in a smooth curvilinear fashion with increasing λ_d_ values. (**B**) The FUI versus CDOM (*a*_440_) values exhibit a crude hyperbolic relationship.

The values of λ_d,hyp_ were related to four water quality parameters (SD, CDOM, CHL, and FUI; Figure 8 and Figure 9). As Figure 8A shows, the SD tended to decrease in a fairly linear fashion as the λ_d,hyp_ increased. The inverse of SD is linearly related to light attenuation coefficients, but a plot of 1/SD versus λ_d_ (not shown) did not yield an obvious relationship. The CDOM values increased very non-linearly as λ_d,hyp_ increased, starting near 560 nm, with most of the increase occurring in a narrow range above λ_d_ = 575 nm (Figure 8B). In contrast, the CHL values peaked around λ_d,hyp_ = ~575 nm Figure 8C). A challenging aspect of the relationship between λ_d_ and water quality parameters is that at longer values of λ_d_ (around 575 nm), both CDOM and CHL may be elevated, and it appears that CDOM and CHL cannot be distinguished based on λ_d_ alone. Multiple regression analyses between λ_d_ and the main optical variables (CHL, CDOM, SM) did not show any useful relationships.

Insofar as λ_d_ and the Forel–Ule index (FUI) are both metrics of visual color (and our FUI were derived from α values that were inversely related to the λ_d_ values), it is not surprising that there is a strong relationship between them. As Figure 9A shows, the FUI increased in a smooth, curvilinear fashion with λ_d_. The FUI is not a continuous variable but rather an integer (classification) variable with a range of 1–21 (hence the leveling off for the two highly (humic) colored lakes, South Sturgeon and Section Eleven, at the top right of the plot). The FUI was also related in a crude hyperbolic fashion to the CDOM, measured as *a*_440_ (Figure 9B). The wide range of CDOM values at high FUI levels reflects the nature of the FUI as a classification variable rather than a continuous variable; even the lowest *a*_440_ values (~3 m^−1^) for FUIs ≥ 17 were for water bodies that were visibly brown. The water bodies with FUI values that were lower than expected for their *a*_440_ value (i.e., those on the right side of the data envelope) generally can be explained by the concomitant presence of high levels of CHL or SM, which tend to cause lower λ_d_ values.

## 5. Discussion

It is evident from Table 2 and Figure S3 that the correction of α is necessary for the accurate calculation of λ_d_ using the Wang method, but as shown by Figure 4, the correction process did not work well for α values < 75° using the Wang method. In contrast, the vdWW method yielded accurate results across most of the λ_d,hyp_ values in the dataset. Consequently, comparisons of the two methods must consider that the Wang method is not corrected for hue angles < 75°, which presents a limitation for this method, especially for datasets such as the MNWI that have large numbers of water bodies with low α values (and high λ_d_ and FUI values, i.e., CDOM-rich water bodies). For example, for the simulated Sentinel-2 dataset used in this paper, 143 observations (44%) were not corrected for the Wang method, but only 15 observations were not corrected for the vdWW method.

For the wavelength ranges within which both methods worked well, the R^2^ values for Sentinel-2 (MSI) and Sentinel-3 (OLCI) were slightly higher for the vdWW method than for the Wang method (Table 2), indicating that the vdWW method had a better statistical fit for these sensors. The R^2^ values were essentially the same for Landsat 8 (OLI). The mean absolute difference (MAD) was lower for the Wang method for the Sentinel-2 sensor and slightly lower for the Landsat 8 sensor, but the vdWW method had a much lower MAD for the Sentinel-3 sensor. Lower MAD values indicate that a method provides results closer to the true values. It is important to note, however, that for both methods the mean absolute percent difference (MAPD) was less than 1%. The vdWW and Wang methods, thus, are both good estimators of λ_d_ over the wavelength ranges applicable for each method.

The sensor-dependent differences between the two methods can be explained as follows. For the vdWW method, the Sentinel-3 data had the highest R^2^ and lowest MAD, most likely because this sensor has the greatest number of visible bands (11) of those used in the calculations. In contrast, only five bands are used for Sentinel-2 and four bands for Landsat 8 [12]. In contrast, for the Wang method, Landsat 8 had the highest R^2^ and lowest MAD. The Wang method uses the three bands from each satellite that are closest to the pre-defined wavelengths. The OLI for Landsat 8 may be the most accurate sensor for the Wang method because it best approximates the reflectance values for the pre-defined wavelengths. Landsat 8 generally has the broadest bandwidths of the bands used in the Wang method, which may partially explain its advantage over the other two satellites.

**Figure 10:**
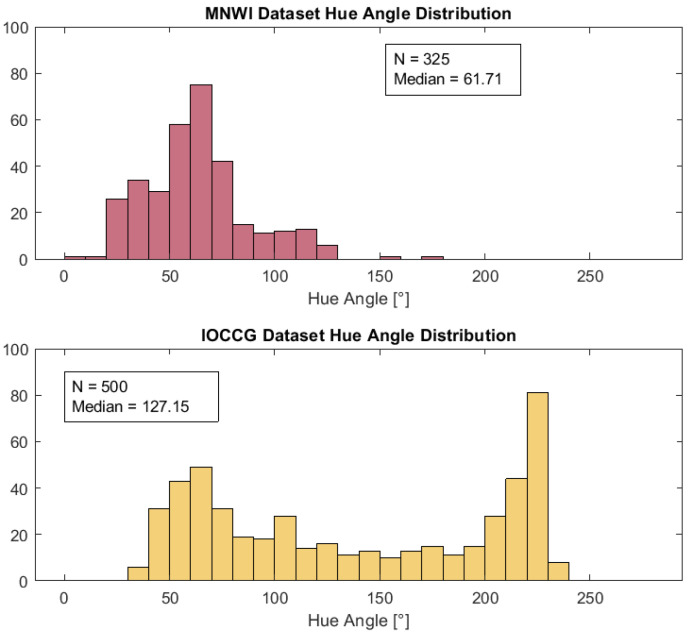
Distribution of hyperspectrally derived hue angles (α) for the MNWI and IOCCG datasets.

A potential explanation for the poor performance of the Wang method at α < 75° is the difference between the MNWI and IOCCG datasets. As the histograms in Figure 10 show, the IOCCG dataset has a broad distribution of samples across the range of α values from 37.1 to 230.8° but relatively few data points for small α values, whereas the MNWI dataset has many more data points in the α range of 30–75° (and fewer at high α values). The differences in data distribution may have led to the correction equation not being a good fit for low hue angles. The vdWW corrections were also derived using the IOCCG data, however, and a poor fit at low α values was not a problem for this method. Thus, the error is related to the particulars of the Wang method. When the α errors (Figure S2) for the Wang method were compared to the α errors for the vdWW method (cf. Figure 4 in [12]), the Wang method had much larger negative α errors, e.g., for the S2 and L8 sensors, an error rate of ~−40° was found for the Wang method at α = 75° versus negligible α errors for the vdWW method. The larger magnitude of α errors for the Wang method likely contributed to the difficulty in applying corrections for this method.

It is appropriate to note that the Wang method was developed primarily to compute FUI values from α values extracted from satellite (MODIS) data [13], and it was applied to a set of 100 global lakes [14] to evaluate the usefulness of the FUI as a metric of the trophic state (degree of eutrophication) of lakes. The set of global lakes and the dataset in that study were reported to include lakes with high CDOM levels, but quantitative CDOM data (e.g., *a*_440_ values) were not reported. The effects of CDOM on α and λ_d_ were not a focus of their study, but the authors did report that some water bodies with FUI values > 10 were influenced by the CDOM as well as the CHL. Although it is difficult to assess the influence of the CDOM on the calculated α values in Wang’s studies [13,14] or to compare the optical distribution of their global lake dataset with that of the MNWI dataset, it is pertinent to note that the MODIS visible sensor bands are very similar to those of Landsat 8, which we noted above had the best performance using the Wang method.

To understand the differences in performance for the two methods, it is necessary to understand the key differences between the methods. Both methods are based on evaluating Equation (2) using the values of reflectance measured by a given satellite sensor. For the vdWW method, all satellite bands with centers between 400 and 710 nm are included, and the evaluation of Equation (2) is specific to the band centers of a given satellite. For the Wang method, only three sensor bands are included, and Equation (2) is evaluated assuming that the bands are close to the pre-defined RGB values. These factors likely led to larger errors in α that were more difficult to correct. Both methods performed comparably for cases where corrections could be applied, but for the Wang method, this constituted only a little more than half of the MNWI dataset, which contains a large number of high-CDOM lakes.

The choice of the method to use to calculate λ_d_ is informed by many factors. Based on our results, corrections to α can be applied more reliably in the vdWW method than the Wang method. The corrections are not trivial; some corrections can have a magnitude of 30–40° (Figure S2). Moreover, if the water bodies being studied include lakes with high λ_d_ (>564.2 nm) and low α (<75°) values, the vdWW method would be more appropriate because the corrections can be applied to more of the dataset. For some applications, however, either method may suffice. When monitoring lakes with known high hue angles, the Wang method may be preferable because it is simpler to implement.

For either method, a few issues are critical to remember. First, if the values of α are not corrected, a non-linear relationship will occur between the “true” and calculated values of λ_d_, i.e., a change in the value of the “true” α will not always result in the same amount of change in the satellite-derived α (see Figure S3). This issue is of concern when changes in the dominant wavelength over time or space are considered. Second, if the α values are not corrected, the derived λ_d_ values may differ significantly from the in situ values.

Finally, regarding the relationships between the water color metrics (e.g., hue) and the optically related water quality variables SD, CHL, CDOM, and SM, we found a generally linear relationship between λ_d_ and SD, which is an “inclusive” metric that depends on all three of the other variables, but no useful relationships were found between λ_d_ and CDOM or CHL. Instead, moderate-to-high values for both metrics were associated with λ_d_ values near 575 nm; λ_d_ alone, thus, cannot distinguish between the CHL and CDOM. As a measure of the hue, the FUI is related to the λ_d_ (Figure 9A) and α; although variables such as the CHL and CDOM affect the hue (and hence FUI), they are quantified in terms of the absorbance at specific wavelengths, which in terms of satellite imagery translates to being quantified in terms of the reflectance in specific wavelength bands. This explains the very crude hyperbolic relationship between the FUI and CDOM (expressed as *a*_440_ in m^−1^) in Figure 9B. The wide ranges of CDOM values at moderate-to-high FUI values reflect the fact that varying amounts of CHL in the water bodies also affect the hue ( and in turn the FUI). A given FUI value can result from various combinations of CDOM and CHL levels. The FUI, a classification variable based on the hue, has only limited capacity to predict actual concentrations of the various optical water quality variables [27], but as Wang et al. [14] demonstrated, it can be used to group lakes into major trophic categories (oligotrophic, mesotrophic, and eutrophic).

## 6. Conclusions

The purpose of this research was to determine the appropriate methods for the remote sensing of water color using the dominant wavelength (λ_d_) metric. Both methods currently used to calculate λ_d_ values from satellite data performed with comparable accuracy in the range where hue angles (α) can be corrected. For the vdWW method, all three satellites that were considered (Sentinel-2, Sentinel-3, and Landsat 8) performed comparably, with Sentinel-3 producing the most accurate results. For the Wang method, the correction of α values <75° was unreliable, and for the three sensors we tested, the method did not yield reliable results for humic-colored water bodies, which tend to have α values < 75°.

## Figures and Tables

**Table 1:** Summary statistics for the dominant wavelength, hue angle, and Forel–Ule index for the in situ hyperspectral data.

	Dominant Wavelength (λ_d,hyp_), nm	Hue Angle (α_hyp_), °	Forel–Ule Index
Mean	567.5	63.3	13
Median	570.7	61.7	13
Std. Dev.	17.1	24.9	3
Min.	493.2	1.8	5
Max.	607.4	173.4	21
25 percentile	565.1	46.7	11
75 percentile	577.3	73.3	15
Number	325	325	325

**Table 2:** Statistical evaluation of λ_d_ values computed using the vdWW and Wang methods compared with λ_d,hyp_ (assumed to be the true values). Statistics were calculated only for the ~180 observations where both methods were corrected.

	λ_d_, vdWW vs. λ_d,hyp_			λ_d_, Wang (corrected) vs. λ_d,hyp_		
	Sentinel-2 MSI	Sentinel-3 OLCI	Landsat 8 OLI	Sentinel-2 MSI	Sentinel-3 OLCI	Landsat 8 OLI
R^2^	0.986	0.997	0.993	0.971	0.966	0.990
MAD *, nm	3.27	0.72	1.84	2.88	2.84	1.77
MAPD *, %	0.59	0.13	0.33	0.52	0.51	0.32
Bias, nm	−3.25	−0.25	−1.67	−2.39	−1.96	−1.55

* MAD = mean absolute difference; MAPD = mean absolute percent difference.

**Table 3:** Coefficients for correction polynomials for the Wang method.

Sensor	c_5_	c_4_	c_3_	c_2_	c_1_	c
Sentinel-2 MSI	−105.25	781.58	−2167.58	2727.22	−1480.20	245.41
Sentinel-3 OLCI	−157.46	1130.01	−3053.26	3784.64	−2061.43	359.95
Landsat 8 OLI	−66.07	488.30	−1324.18	1588.93	−789.49	106.97

## Data Availability

The Wisconsin in situ spectra and some of the Minnesota spectra are available in the Gloria dataset [32]. All Minnesota in situ spectra and the data derived from them for the α, λ_d_, FUI, and associated water quality data are available from the Data Repository for U of MN (DRUM) at: https://hdl.handle.net/11299/250566.

## Supplementary Materials

The following supporting information can be downloaded at: https://www.mdpi.com/article/10.3390/s23031071/s1. Figure S1: Non-linear inverse relationship between the hue angle, α, and dominant wavelength, λ_d_, as exemplified by computed values for α and λ_d_ from the 325 hyperspectra used in this study. Figure S2: Plots of λ_d_ values computed for three satellite sensors using the Wang [13] method and uncorrected α values: (A) S-2 MSI; (B) S-3 OLCI; (C) OLI. Figure S3: Hue angle errors of the three satellite sensors for the Wang method applied to the IOCCG dataset. Table S1: Summary of water quality data for water bodies in Figure 5 with reflectance spectra characteristics of various optically dominant variables.
