# Supplementary material for: Comparison of Two Water Color Algorithms: Implications for the Remote Sensing of Water Bodies with Moderate to High CDOM or Chlorophyll Levels
Source: Sensors (Basel). 2023 Jan 17;23(3):1071. doi: 10.3390/s23031071 (PMC9920161; doi:10.3390/s23031071)
Supplement: Supplementary file 1 [file sensors-23-01071-s001.zip › sensors-2130204-supplementary.pdf]

Supplemental Information

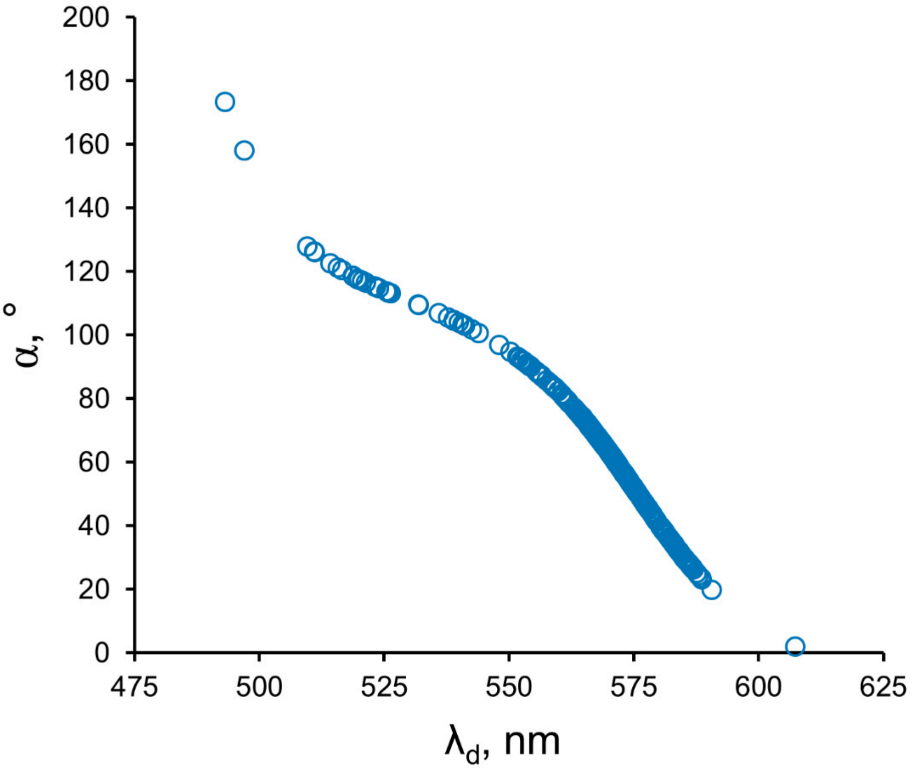

Figure S1. Nonlinear, inverse relationship between hue angle, α, and dominant wavelength, λ_d, as exemplified by computed values for α and λ_d from the 325 hyperspectra ued in this study.

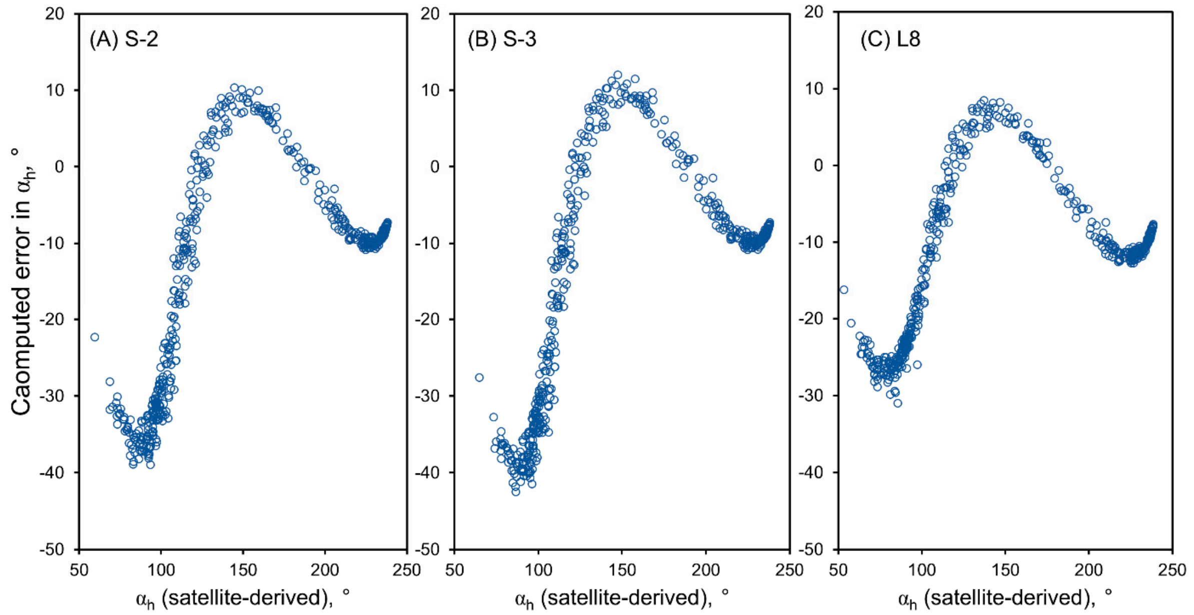

Figure S2. Plots of λ_d computed for three satellite sensors by the Wang [13] method using uncorrected α values: (A) S-2 MSI, (B) S-3 OLCI, and (C) OLI.

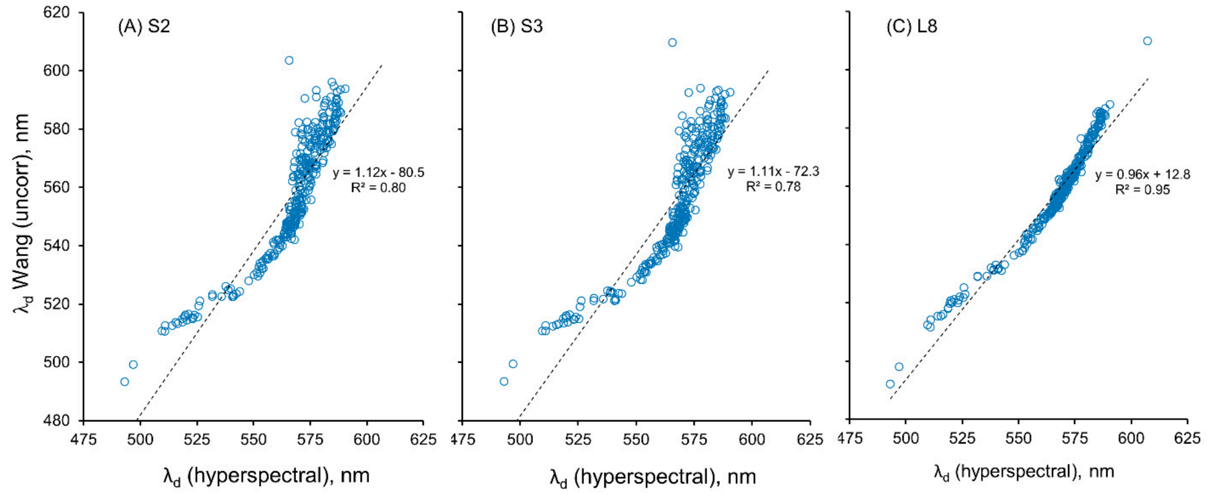

Figure S3: Hue angle error of the three satellite sensors for the Wang method applied to the IOCCG dataset.

Table S1. Summary of water quality data for water bodies in Figure 5 with reflectance spectra characteristic of various optically dominant variables.

Water Body*	Dominant variable	Secchi depth m	Chlorophyll mg/m³	CDOM, a_{440} m^{-1}	Suspended matter mg/L
Sabin L.	water	19.2	0.27	0.05	2.4
Woman L.	water + CHL	2.4	2.45	0.78	6.9
Halstead's Bay	CHL	0.5	49.8	1.5	14.6
Johnson Bog	CDOM	0.5	6.5	27.9	2.4
Section Eleven	CDOM	0.5	2.9	23.5	8.0
Big Sandy L.	CDOM + CHL	1.0	27.5	9.9	8.8
Flowage L.	CDOM + SM	0.9	9.7	22.6	10.5
St. Louis R.	SM + CDOM	--	2.5	11.0	145
